# Investigation of the analgesic efficacy of ultrasound-guided thoracolumbar interfacial plane block in vertebral surgery: A prospective randomized clinical study

**DOI:** 10.15537/smj.2022.43.10.20220467

**Published:** 2022-10

**Authors:** Hakan Tapar, Özgür Demir, Ali Genç, Mehtap G. Balta, Vildan Kölükçü, Tugba Karaman, Serkan Dogru, Serkan Karaman, Mustafa Suren

**Affiliations:** *From the Department of Anesthesiology and Reanimation (Tapar, Balta, Kölükçü, T. Karaman, S. Karaman,); from the Department of Neurosurgery (Demir), Medical Faculty, Tokat Gaziosmanpasa University, from the Department of Anesthesiology and Reanimation (Genç), Turhal State Hospital, from the Department of Anesthesiology and Reanimation (Suren), Medical Faculty, Samsun University, Tokat, and from the Department of Anesthesiology and Reanimation (Dogru), Mersin City Hospital, Mersin, Turkey.*

**Keywords:** nerve block, spine, postoperative period, pain, tramadol, morphine, analgesia, visual analog scale

## Abstract

**Objectives:**

To investigate the effect of thoracolumbar interfacial plane block (TLIP) on analgesic consumption and pain score in vertebral surgery.

**Methods:**

All patients (64 patients undergoing vertebral surgery) were randomly allocated as Group T (patients with block, n=32) and Group C (patients without block, n=32). After surgery, patient-controlled analgesia using tramadol was administered to all patients. Pain intensity was evaluated with visual analogue scale (VAS; recovery room at 1, 2, 6, 12, and 24 hours postoperative), and as rescue analgesia, morphine was administered to patients with VAS scores of >4. In this study, total tramadol consumption, the number of patients requiring morphine, VAS score, and Quality of Recovery-40 of all patients questionnaire was evaluated.

**Results:**

There were important differences between the 2 groups according to mean postoperative tramadol consumption (Group T and Group C; 180 mg [100-260] vs. 210 mg [100-300]; *p*=0.001) and the number of patients requiring additional analgesia (n=4; 12.5% vs. n=24; 75%, *p*=0.000). There were important differences between the 2 groups according to the postoperative VAS pain score (*p*=0.000).

**Conclusion:**

Ultrasound-TLIP reduces analgesic consumption and pain severity after vertebral surgery. Therefore, it is an important regional analgesia technique.

**ClinicalTrials.gov Grant No.:**

**NCT04548076**

**V**ertebral surgeries are common surgical procedures, and lumbar instrumentation surgery is especially associated with significant pain and immobility.^1^ Pain in vertebral surgery may arise from the skin, muscle, tissue trauma, and surgical incision.^2^ Insufficient pain control can reduce patient satisfaction, chronic pain, and prolong hospital stays. Therefore, efficient and safe methods of administering postoperative analgesia for the achievement of early recovery and better outcomes is essential.^3^ Nonsteroidal anti-inflammatory drugs, paracetamol and opioids are used for pain. In addition, regional anaesthesia techniques (namely, neuraxial anaesthesia and local anaesthetic infiltration) are used to reduce the nausea, vomiting, and constipation associated with opioids and also for pain control.

Thoracolumbar interfascial plane (TLIP) block has been used in recent years.^4^ This block can be easily applied with a low risk of complications.^5^ Thoracolumbar interfascial plane block can be carried out via 2 different approaches; the classical (cTLIP) technique and the modified (mTLIP) technique with ultrasound guidance. The cTLIP technique involves the administration of local anaesthetic between the multifudus and longisumus muscles, and the mTLIP technique administers it between the longisumus and iliocastalis muscles.

In this study, the effect of TLIP block on analgesic consumption and pain score in vertebral surgery was investigated.

## Methods

**Figure 1:**
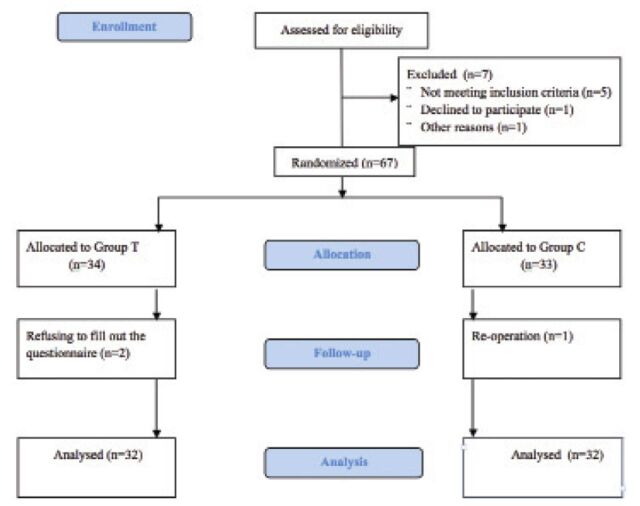
- Consolidated Standards of Reporting Trials flow diagram.

It is a study carried out as a prospective, randomized in patients planned vertebral surgery between September 2020 and August 2021 at Tokat Gaziosmanpasa University Hospitals, Tokat, Turkey. Local Ethics Committee of Tokat Gaziosmanpasa University, Tokat, Turkey, (20-KAEK-076) approval was obtained. All patients provided written informed consent for this study and the Declaration of Helsinki was complied with. The inclusion criteria were a total of 64 patients (18-65 years), that were scheduled for lumbar disc, and 2-, 3- or 4-levels of posterior lumbar instrumentation surgery, with a physical status American Society of Anesthesiologists I-III. Patients with chronic pain, preoperative analgesic consumption, bleeding diathesis, anticoagulant, or corticosteroid use with patients who canceled the procedure and refused further participation were excluded. The study was completed with 64 patients (Group T, n=32 and Group C, n=32; Figure 1).

**Figure 2:**
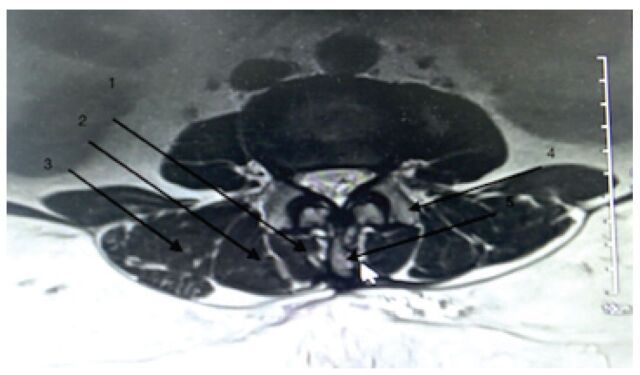
- Magnetic resonance anatomy for Thoracolumbar interfascial plane block. 1) Multifudus muscles; 2) longisumus muscles; 3) iliocastalis muscles; 4) transverse process; 5) spinous process

With all patients, general anaesthesia was induced with 2 mg/kg propofol (Dormofol, Istanbul/Turkey) 1 μg/kg fentanyl (Talinat, Istanbul/Turkey) and 0.6 mg/kg rocuronium bromide (Esmeron, Istanbul/Turkey). Anesthesia was continued with sevoflurane (Sojourn, Istanbul/Turkey) (1 MAC) and 50/50 oxygen/air. After general anesthesia in the group TLIP(+), a 20 mL mixture of 0.25% bupivacaine (Marcaine, Istanbul/Turkey) and 1% lidocaine (Aritmal, Istanbul/Turkey) was injected bilaterally between the longissimus and multifidus muscles at the third lumbar vertebra (L3) in the prone position with a 100 mm - 20-G needle (Vygon) under the guidance of a high linear probe of the ultrasound system (Hitachi Aloka Noblus, Tokyo, Japan, Figure 2). Thoracolumbar interfascial plane block was not applied to the patients in the control group.

After surgery, all patients were fitted with a patient-controlled analgesia device that contained tramadol hydrochloride (Tradolex, Ankara/Turkey) and the device was set up such that there was a 20 mg bolus dose, a 10-minute lock time, and a maximum of 3 doses per hour. Again, 10 mg/kg paracetamol (Partemol, Istanbul/Turkey) every 8 hours was routinely prescribed to all patients. The VAS score for pain was evaluated in the recovery room at 1, 2, 6, 12, and 24 hours. For patients with a VAS score of >4, a rescue analgesic (0.03 mg/kg intravenous morphine (Morfin Hidroklorür, Istanbul/Turkey) was administered. The Quality of Recovery-40 (QoR-40) questionnaire was completed to evaluate patient satisfaction at the 24^th^ postoperative hour. Total consumption of tramadol and the VAS values were recorded for all patients.

A pilot study revealed that the total tramadol consumption of patients who did not undergo TLIP was found to be 240±50 mg. Assuming a 15% reduction in tramadol consumption in patients undergoing TLIP (a power of 80% [beta=0.2]) with a 5% significance level (alpha=0.05), 60 patients were required to detect a statistically significant difference.

### Statistical analysis

The Statistical Package for the Social Sciences, version 20.0 (IBM Corp., Armonk, NY, USA) was used. The normality was evaluated using the Kolmogorov-Smirnov test. The Mann-Whitney-U test, Pearson’s chi-squared test, or independent samples t-test were carried out to compare the data. A *p*-value of <0.05 was considered significant.

## Results

A total of 64 patients (Group T, n=32 and Group C, n=32) were analysed in this study. From the Group T, 2 patients refused to fill out the questionnaire and from the Group C one patient re-operated and they were excluded. The data regarding the patients and surgery are presented in Table 1.

**Table 1:** - Patients’ demographics and surgical data.

Variables	Group T	Group C	*P*-values
***Gender***			
Female	17 (53.1)	13 (40.6)	0.316
Male	15 (46.9)	19 (59.4)	
Age (year), mean±SD	50.03±12.286	51.13±11.290	0.712
BMI (kg/m^2^), mean±SD	27.641±5.284	28.526±4.383	0.468
***ASA score***			
I	3 (9.4)	7 (21.9)	
II	25 (78.1)	19 (59.4)	0.244
III	4 (12.5)	6 (18.8)	
***Surgery type***			
Spinal instrumentation	16 (50.0)	13 (40.6)	0.451
Lumbar discectomy	16 (50.0)	19 (59.4)	
Surgical duration (minute), median (min-max)	193 (120-360)	180 (90-420)	0.709

Values are presented as a number and precentage (%). SD: standard deviation, BMI: body mass index, ASA: American society of anesthesiologist, min: minimum, max: maximum

The postoperative VAS pain score was statistically lower in Group T (*p*=0.000; Table 2). The quality of postoperative patient recovery was similar between groups. The QoR-40 median score was 181 (135-197) in Group T and 186 (135-197) in Group C (*p*=0.532; Table 2).

**Table 2:** - The postoperative visual analogue scale and Quality of Recovery-40 scores of the patients.

Variables	Group T	Group C	*P*-values
***Postoperative VAS***			
One hour	2 (0-4)	4 (1-8)	
2 hours	2 (0-4)	4 (2-7)	
6 hours	1 (0-4)	3 (1-7)	0.000*
12 hours	1 (0-3)	2 (0-5)	
24 hours	0 (0-2)	2 (0-5)	
***QoR-40 total score***	181(135-197)	186 (135-197)	0.532
PC	53 (41-60)	53 (35-60)	0.946
ES	41.5 (30-45)	44 (28-47)	0.116
PI	23.5 (9-25)	24 (5-25)	0.927
PS	34 (24-35)	34.5 (28-35)	0.045
P	32 (23-34)	31.5 (21-35)	0.962

Values are presented as a median (minimum-maximum). The Mann-Whitney-U test was used for statistical analyze. *statistically significant, VAS: visual analogue scale, QoR-40: quality of recovery-40, PC: physical comfort, ES: emotional state, PI: physical independence, PS: patient support, P: pain

Postoperative tramadol consumption and the number of additional analgesia were found to be lower in Group T (180 mg [100-260] vs. 210 mg [100-300]; *p*=0.001, 12.5% vs. 75%; *p*=0.000; Table 3).

**Table 3:** - The postoperative analgesic requirements of the patients.

Requirements	Group T	Group C	*P*-values
Postoperative tramadol consumption (mg), median (minimum-maximum)	180 (100-260)	210 (100-300)	0.001*
Additional analgesic requirement (mg), mean±SD	0.375±1.008	3.975±2.895	0.000*
***Additional analgesic requirement, n (%)***			
No	28 (87.5)	8 (25.0)	0.000*
Yes	4 (12.5)	24 (75.0)	

The Mann-Whitney-U test and Pearson Chi-square test were used for statistical analyze. *statistically significant, SD: standard deviation

There was not difference between the groups according to postoperative nausea (*p*=0.43) and vomiting (*p*=0.545; Table 4).

**Table 4:** - The postoperative nausea and vomiting scores of the patients.

Variables	Group T	Group C	*P*-values
***Nausea***			
No	22 (68.8)	19 (59.4)	0.434
Yes	10 (31.2)	13(40.6)	
***Vomiting***			
No	26 (81.3)	24 (75.0)	0.545
Yes	6 (18.9)	8 (25.0)	

Variables are presented as a number and precentage (%). The Pearson Chi-square test was used for statistical analyze.

## Discussion

Thoracolumbar interfascial plane block significantly reduces postoperative tramadol consumption, morphine requirement for rescue analgesia, and VAS pain score within 24 hours following vertebral surgery. It is hence an important regional analgesia technique for vertebral surgery.

Vertebral surgeries are common surgical procedures that are being carried out increasingly frequently. Patients undergoing such surgeries may suffer from moderate to severe pain.^6^ The standard analgesia protocols for spine surgery usually include opioids for adequate pain management. High-dose opioids can be used in these patients, but they can result in side effects such as nausea, vomiting, and constipation. Therefore, alternatives analgesic methods are important for both reducing opioid requirements and promoting the early mobilization of patients.^7,8^

Thoracolumbar interfascial plane block is a type of interfascial block and at the L3 level have an analgesia that spreads from L1-S1.^9^ In this study, we found that the TLIP block decreased patient consumption of opioids and provided adequate analgesia. There are similar prospective studies that reported similar results. Ammar et al^10^ used a 20 mL mixture of 0.25% bupivacaine and 1% lidocaine on each side, and Ueshima et al^11^ used 20 mL of 0.375% levobupivacaine on each side. According to these study, TLIP block provided effective analgesia after vertebral surgery.

We found that the requirement for morphine for rescue analgesia was reduced in the TLIP group. In a study of lumbar fusion surgery patients, an average of 23 mg of morphine was administered in patients who did not undergo TLIP, but not required in patients who underwent TLIP.^12^

The VAS pain scores in the postoperative period were significantly lower in the TLIP group, which is in line with other studies showing similar results. In patients who underwent lumbar spine fusion surgery, the VAS pain scores for patients at movement and at rest were significantly lower compared to patients who did not receive TLIP block.^13^ The same differences in VAS pain scores were also noted in a different study involving lumbar disc surgery.^14^

The Quality of Recovery-40 is used to evaluate the quality of healing. In one of these studies, the quality of recovery and the patient’s postoperative health status were better in the TLIP group, but we failed to see such a difference in our study.^15^ This could be explained by the fact that VAS pain values were generally ≤4 in both groups and that there were no differences in nausea and vomiting between the groups.

### Study limitations

First, groups has heterogeneous patient scheduled lumbar disc and lumbar instrumentation surgery. Thus, planning a study for only one indication might be beneficial. Second, this prospective study has a relatively small sample size. Finally, because of general anesthesia, it was not detected whether or not there was a lost sensory area after the block procedures.

In conclusion, bilateral TLIP block could provide sufficient analgesia and significantly reduce patient opioid consumption after vertebral surgery. Therefore, it is an important regional anesthesia technique that can be used for multimodal analgesia.
